# TransAnnot—a fast transcriptome annotation pipeline

**DOI:** 10.1093/bioadv/vbae152

**Published:** 2024-10-22

**Authors:** Mariia Zelenskaia, Yazhini Arangasamy, Milot Mirdita, Johannes Söding, Venket Raghavan

**Affiliations:** Quantitative and Computational Biology, Max-Planck Institute for Multidisciplinary Sciences, Göttingen 37077, Germany; Quantitative and Computational Biology, Max-Planck Institute for Multidisciplinary Sciences, Göttingen 37077, Germany; School of Biological Sciences, Seoul National University, Seoul 08826, South Korea; Quantitative and Computational Biology, Max-Planck Institute for Multidisciplinary Sciences, Göttingen 37077, Germany; Campus-Institut Data Science (CIDAS), Göttingen 37077, Germany; Quantitative and Computational Biology, Max-Planck Institute for Multidisciplinary Sciences, Göttingen 37077, Germany; Institute for Medical Informatics, Statistics and Epidemiology, University of Leipzig, Leipzig 04107, Germany

## Abstract

**Summary:**

The annotation of deeply sequenced, *de novo* assembled transcriptomes continues to be a challenge as some of the state-of-the-art tools are slow, difficult to install, and hard to use. We have tackled these issues with TransAnnot, a fast, automated transcriptome annotation pipeline that is easy to install and use. Leveraging the fast sequence searches provided by the MMseqs2 suite, TransAnnot offers one-step annotation of homologs from Swiss-Prot, gene ontology terms and orthogroups from eggNOG, and functional domains from Pfam. Users also have the option to annotate against custom databases. TransAnnot accepts sequencing reads (short and long), nucleotide sequences, or amino acid sequences as input for annotation. When benchmarked with test data sets of amino acid sequences, TransAnnot was 333, 284, and 18 times faster than comparable tools such as EnTAP, Trinotate, and eggNOG-mapper respectively.

**Availability and implementation:**

TransAnnot is free to use, open sourced under GPLv3, and is implemented in C++ and Bash. Source code, documentation, and pre-compiled binaries are available at https://github.com/soedinglab/transannot. TransAnnot is also available via bioconda (https://anaconda.org/bioconda/transannot).

## 1 Introduction

Transcriptome annotation is the task of associating biological and functional context to RNA-Seq data. This represents an essential step in rendering the data usable, especially in cases when a reference genome is absent (Raghavan *et al.* 2022). The process typically uses protein sequences, translated *in silico* from pre-assembled transcripts, as inputs/queries. These query sequences are then compared against databases of well-annotated proteins and/or orthogroups thereof using a combination of sequence-sequence and sequence-profile searches to establish homology. Annotations are then transferred over to the queries on the basis of shared sequence features (e.g., sufficient sequence similarity) between the queries and their matches. This typically results in the identification of closely-related homolog(s) from well-studied species, identification of orthologous groups, assignment of Gene Ontology (GO; Ashburner *et al.* 2000) identifiers (for cellular components, molecular function, and biological process), and recognition of functional signatures such as domains and active sites. A handful of software suites have been published over the years to facilitate transcriptome annotation, e.g., Annocript (Musacchia *et al.* 2015), Blast2GO (Conesa *et al.* 2005), dammit (Scott *et al.* 2019), EggNOG-mapper (Cantalapiedra *et al.* 2021), EnTAP (Hart *et al.* 2020), and Trinotate (Bryant *et al.* 2017). However, almost all of these suffer from a reliance on multiple, slow sequence search tools including BLAST (Camacho *et al.* 2009) and HMMER3 (Eddy 2011), and expect the user to not only procure the search databases but also manage the often complicated installation process and dependencies. A number of these tools (e.g., dammit) have also fallen out of maintenance over the years (Supplementary Material S1 Table III). As such, the task of transcriptome annotation with existing tools is a cumbersome endeavour for end users who are increasingly biologists with a limited computing background.

Here, we present TransAnnot, a fast, easy to use transcriptome annotation tool that addresses the aforementioned challenges. It leverages the fast-yet-sensitive protein sequence-to-sequence/sequence-profile searches and in-built databases offered by MMseqs2 (Steinegger and Söding 2017) to identify homologs from Swiss-Prot (The UniProt Consortium 2023), assign orthogroups and GO terms from eggNOG DB (Huerta-Cepas *et al.* 2019), and identify domains using Pfam (Mistry *et al.* 2021) respectively. It also offers the option to assemble sequencing data internally with the direct-to-protein sequence assembler PLASS (Steinegger *et al.* 2019) (although only for reads > = 100 nt). With minimal dependencies and a modular construction, TransAnnot is easy to install, fast to execute, and easy to use. While TransAnnot requires pre-translated (i.e., protein) sequences as inputs, it can optionally operate directly upon nucleotide sequences (albeit with a large performance penalty). TransAnnot also offers the option to optionally cluster the inputs to reduce redundancy before annotation. TransAnnot can also handle long read RNA-Seq data. Command invocation is as simple as transannot annotate < paths_to_files > once the mandatory databases (EggNOG DB, Swiss-Prot, and Pfam have been downloaded). Annotation against user-defined databases is also possible. Benchmarking with translated sequence sets from a variety of prokaryotic and eukaryotic organisms demonstrated TransAnnot possessing sensitivity comparable to the state of the art (mean annotation rate of > 66%) while being significantly faster (between 9 and 333 times) than tools such as EggNOG-mapper, EnTAP, and Trinotate.

## 2 Materials and methods

TransAnnot is a command line utility constructed in a modular fashion. The primary module of interest to the end user is the eponymously named annotate module whose functionality is described hereonforth (unless explicitly stated otherwise).

### 2.1 Input

The primary input is sequencing data provided either as FASTQ-formatted file(s) of raw sequencing reads or as a FASTA-formatted file of amino acid sequences, translated from a transcriptome assembled using an external tool (e.g., TransDecoder (Haas 2018)). If the user inputs sequenced reads, TransAnnot uses PLASS (Steinegger *et al.* 2019) to assemble them into protein sequences which are then used as queries for annotation. However, PLASS has not yet been extensively benchmarked for transcriptome assembly and requires a minimum read length of >=100nt. Further, although TransAnnot can accept nucleotide sequences as inputs the run time becomes unacceptably large (on the order of multiple hours to days). Therefore we strongly recommend supplying translated amino acid sequences as inputs. Depending upon the TransAnnot module invoked, the user must also specify some subset of the integrated target databases and (optionally) values that affect the sequence searches. Custom databases to be annotated against can also be supplied after having been converted into the MMSeqs2 format.

### 2.2 Algorithm

**Figure 1.**
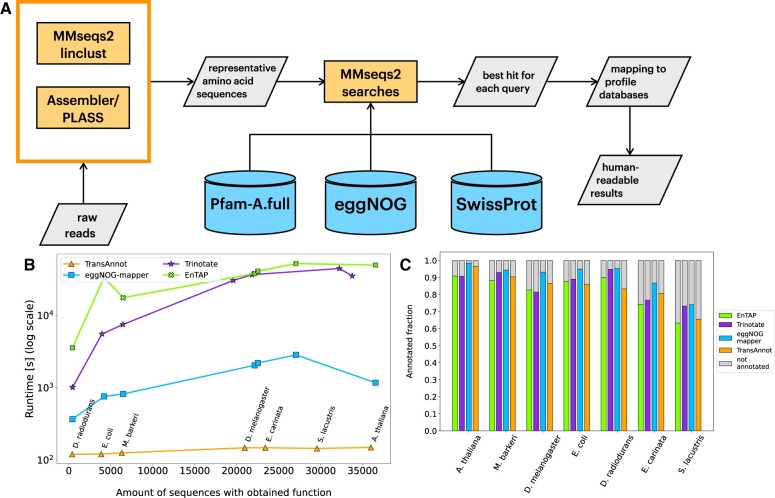
(A) TransAnnot optionally assembles raw reads into translated amino acid sequences using our protein-level assembler (PLASS; Steinegger *et al.* 2019). Alternatively, amino acid sequences translated from an external assembly can be supplied; although the tool accepts nucleotide sequence sets as inputs this is not recommended as of the current version due to performance considerations. Optionally, the input sequences are clustered in linear time to extract representative sequences for annotation. MMseqs2 is then used to search the (representative) sequences against Pfam, eggNOG DB, and Swiss-Prot (default databases) or against a set of used-supplied databases. For the sequence database (SwissProt) targets, only the best hit is retained. For the profile database targets, full coverage of each query is ensured by preserving non-overlapping hits. (B) Comparison of runtimes (in log(seconds)) as a function of the number of annotated sequences. (C) Proportions of successfully annotated sequences for TransAnnot and contemporaries. A sequence is considered annotated if it shares at least 50% sequence identity with its match and has a match in at least one of the three default target databases.

The default annotation process consists of three steps: (1) the input sequences are converted into MMseqs2 database format and clustered in linear time using the linclust module from MMseqs2 (Steinegger and Söding 2018). This step can be beneficial as transcriptomes, especially ones assembled *de novo*, tend to be heavily redundant, featuring multiple transcript isoforms for each captured (and expressed) genomic locus. This redundancy may be undesirable in some situations, e.g., when using the transcriptomic data to establish a gene catalogue by proxy (Raghavan *et al.* 2022). Clustering can be disabled should the user wish to retain all transcript isoforms (e.g., for downstream differential isoform expression analysis). (2) Each (representative) sequence is then searched against the Swiss-Prot protein sequence database, orthogroup profiles from the eggNOG DB, and functional domain profiles from Pfam (Pfam-A.full); all three are formatted as MMseqs2 sequence and profile databases respectively. Swiss-Prot was chosen to identify homologs amongst its high-confidence, manually curated protein sequences. EggNOG DB was included to facilitate identification of orthogroups and GO terms. Pfam was included as a standard source of functional domain annotations (Fig. 1A). TransAnnot, by default, only accepts hits with *E*-values < 0.00001. TransAnnot also offers the annotatecustom module to facilitate annotations against user-supplied databases. To ensure full coverage of query, non-overlapping hits are retained for the targets from profile DBs.

### 2.3 Output

A tab-separated text file comprising query IDs, target IDs from Swiss-Prot, and mapped IDs from Pfam and eggNOG along with columns for *E*-value, sequence identity, and bit score for the match. Depending on the number of annotation sources, a transcript can have more than one line in the annotation output.

## 3 Results

### 3.1 Benchmarking

We used seven *de novo* assembled transcriptomes representing all kingdoms of life. We pre-processed the data with fastp v0.22.0 (Chen *et al.* 2018) and assembled the reads using Trinity v2.13.2 with default parameters (Grabherr *et al.* 2011). We used Trinity in particular as it generates, by default, the gene-transcript mapping file required by Trinotate, a transcriptome annotation suite we wished to benchmark against. Translated amino acid sequences were obtained using TransDecoder v5.5.0. We disabled clustering in TransAnnot (—no-run-clust) and used a very permissive sequence similarity threshold (—min-seq-id 0.3) to ensure that the results are comparable as an equivalent feature for clustering did not exist in all the other tools benchmarked alongside. We compared TransAnnot’s core annotate module (version Git: 3-e15e316) against eggNOG-mapper v2.1.9, EnTAP v0.10.8, and Trinotate v3.2.2 as these are the most used transcriptome annotation tools available that are open source and free to use. The tools were benchmarked with 128 CPU-cores and 4GB RAM per CPU on a server featuring 2× AMD EPYC 7742 64-core processors, 1TB system memory, and Rocky Linux 8.6.

### 3.2 Annotated fraction

TransAnnot annotated 66–96% of the transcriptome(s) from seven model organisms. This fraction of transcriptome annotated is similar or better to that of Trinotate, eggNOG-mapper, and EnTAP (Fig. 1C, Supplementary Material S2).

### 3.3 Run time

TransAnnot is consistently and considerably faster than the other tools. It took between 120 and 149 seconds on the tested data sets containing 907–116 830 sequences (Fig. 1B). The average run time was 223, 146 and 10 times faster than EnTAP, Trinotate and eggNOG mapper respectively across all tested species (Fig. 1B).

### 3.4 Annotation of long read data

Long reads can also be given as input to TransAnnot using ‘easytransannot’, ‘annotate’ and ‘annotatecustom’ modules. With ‘easytransannot’, PLASS assembles long reads and the assembled sequences are annotated by TransAnnot. For paired end input, both files from samples must be provided. For single end input, reads from samples should be concatenated into a single file before annotation. The ‘annotate’ and ‘annotatecustom’ modules directly subjects long-reads to translated searches. Some example runs and results can be found in Supplementary Material S1 Section II Benchmarking.

## 4 Conclusion

TransAnnot is able to annotate transcriptomes multitudes faster at a sensitivity competitive with established annotation tools and is also input agnostic with regard to long read/short read sequencing data and nucleotide/amino acid sequences. Its most important advantages are its simple and fast installation with minimal dependencies on external tools, its modularity, and plug-and-play user-friendliness.

## Supplementary Material

vbae152_Supplementary_Data

## Data Availability

SRA identifiers for the RNA-Seq data used for benchmarking are indicated in Supplementary Material S1 Table I.
