# Supplementary material for: TransAnnot—a fast transcriptome annotation pipeline
Source: Bioinform Adv. 2024 Oct 22;4(1):vbae152. doi: 10.1093/bioadv/vbae152 (PMC11530227; doi:10.1093/bioadv/vbae152)
Supplement: vbae152_Supplementary_Data [file vbae152_supplementary_data.pdf]

Supplementary Material
TransAnnot - a fast transcriptome annotation pipeline

Zelenskaia M.,[1] Arangasamy Y.,[1] Mirdita M.,[2] Söding J.,[1,3] and Raghavan V.[1,4]

[1] *Quantitative and Computational Biology, Max-Planck Institute for Multidisciplinary Sciences, Göttingen, Germany*
[2] *School of Biological Sciences, Seoul National University, Seoul, South Korea*
[3] *Campus-Institut Data Science (CIDAS), Göttingen, Germany.*
[4] *Institute for Medical Informatics, Statistics and Epidemiology, University of Leipzig, Leipzig, 04107, Germany*

I. BENCHMARKING DATA

All data were 150 bp paired-end bulk RNA-Seq samples sequenced on Illumina instruments.

TABLE I. Species and SRA accessions of benchmarking data.

Name	SRA accession
Arabidopsis thaliana	SRR22017537
Drosophila melanogaster	SRR20326858
Deinococcus radiodurans	SRR19025978
Elaphe carinata	SRR9942934
Escherichia coli	SRR19854868
Methanosarcina barkeri	SRR13089367
Spongilla lacustris	SRR1168575

II. BENCHMARKING

Command modifications: Values of parameters/inputs that deviated from those set as defaults and/or suggested by tool authors have been indicated below. Tools were run following recommendations in respective documentations. If a command is not mentioned below, that tool/sub-tool was run with default values for all parameters. Parameters for accepting common input files (e.g., FASTA files) are also not indicated below except for TransAnnot. Specific databases, when used, are indicated in angle brackets; default databases as recommend by tool authors used unless specified. (Scripts available in the TransAnnot GitHub repository.)

fastp (reads pre-processing):
```
fastp --detect_adapter_for_pe --correction --average_qual=20
--qualified_quality_phred=20 --n_base_limit=0
--length_required=35;
```

Trinity (assembly):
```
Trinity --seqType fq --full_cleanup --verbose --max_memory
2̈40G¨-bflyCalculateCPU --CPU 32;
```

TransDecoder (*in silico* protein sequence prediction):
```
diamond blastp --ultra-sensitive --db <uniprot_uniref90_db>
--max-target-seqs 1 --outfmt 6 --evalue 1e-5 --threads 32;
TransDecoder.Predict --single_best_only --retain_blastp_hits
<diamond_output_from_above>;
```

eggnog-mapper:
```
--itype proteins --clean_overlaps all --report_no_hits
--pfam_realign realign --go_evidence all --report_orthologs
--sensmode ultra-sensitive -m diamond --cpu 128;
```
All default databases downloadable for eggnog-mapper were used.

EnTAP:
```
EnTAP --runP --ini <config_file> -d <ncbi_nr_db> -d
<swissprot_db> -d <trembl_db> -t 128;
```

Trinotate:
```
blastx -db <swissprot_db> -num_threads 128 -max_target_seqs
1 -outfmt 6 -evalue 1e-5;
blastp -db <swissprot_db> -num_threads 128 -max_target_seqs
1 -outfmt 6 -evalue 1e-5;
hmmscan --cpu 128 --domtblout <pfam_out>;
signalp6 --organism other --write_procs 128 --format none
--mode fast;
tmhmm --short --noplot;
<path_to_util/extract_GO_assignments_from_Trinotate_xls.pl>
-T --include_ancestral_terms;
```

TransAnnot:
```
transannot createquerydb <input_fasta_file> <query_db_name>
<query_db_tmp_dir_name>;  transannot annotate <query_db_name>
<pfam_db> <eggnog_db> <swissprot_db> <results_file_name>
<tmp_dir_name> --min-seq-id 0.3 --no-run-clust;
```

Parsing of results: A query was considered as annotated if the target hit shares at least 50.0% sequence identity and has at least one annotation mapped. For eggnog-mapper, the results were filtered with a minimum E-value of 10^{-10}.

Annotating long-reads input: We tested TransAnnot on annotating long-reads RNA and DNA sequences. For the test runs, a sample long-reads RNA sequences from human embryonic stem cells was taken (Study ID: SRP518114, Run ID: SRR29713566, single-end). For long-reads DNA sequences, a few runs on genomic sequencing of *Staphylococcus pseudintermedius* isolates from canines were taken (Study ID:SRP518114, Run IDs: SRR27301225, SRR27301226, SRR27301227 and SRR27301233, single-end). Annotation results are provided here `https://github.com/soedinglab/transannot/tree/main/examples/longreads_result`.

Long-reads RNA sequence input:
```
transannot easytransannot SRR29713566.fastq <pfam_db>
<eggnog_db> <swissprot_db> <longreads_output>
<tmp_dir_name>;
```
(or)
```
transannot createquerydb SRR29713566.fastq SRR29713566_db
<tmp_dir_name>;
transannot annotate SRR29713566_db <pfam_db> <eggnog_db>
<swissprot_db> <longreads_output> <tmp_dir_name>;
```

Long-reads DNA sequence input:
```
cat SRR27301225.fastq SRR27301226.fastq SRR27301227.fastq
SRR27301233.fastq > single-end-reads.fastq
transannot easytransannot single-end-reads.fastq
<pfam_db> <eggnog_db> <swissprot_db> <longreads_output>
<tmp_dir_name>;
```

III. SOFTWARE VERSIONS

TransAnnot Git: 3-e15e316, Trinotate v3.2.2 [1], eggnog-mapper v2.1.4-2 [2], EnTAP v0.10.8 [5], Trinity v2.13.2 [4], TransDecoder v5.5.0.

[1] Bryant, D.M. et al (2017). A tissue-mapped axolotl de novo transcriptome enables identification of limb regeneration factors. *Cell Reports*, **18**(3), 762–776.

[2] Cantalapiedra, C.P. et al (2021a). eggNOG-mapper v2: Functional Annotation, Orthology Assignments, and Domain Prediction at the Metagenomic Scale. *Molecular Biology and Evolution*, **38**(12), 5825–5829.

[3] Cantalapiedra, C.P. et al (2021b). eggNOG-mapper v2: Functional Annotation, Orthology Assignments, and Domain Prediction at the Metagenomic Scale. *Molecular Biology and Evolution*, **38**(12), 5825–5829.

[4] Grabherr, M.G. et al (2011). Full-length transcriptome assembly from RNA-seq data without a reference genome. *Nature Biotechnology*, **29**, 644–652.

[5] Hart, A.J. et al (2020). Entap: Bringing faster and smarter functional annotation to non-model eukaryotic transcriptomes. *Molecular Ecology Resources*, **20**(2), 591–604.

[6] Musacchia, F. et al (2015). Annocript: a flexible pipeline for the annotation of transcriptomes able to identify putative long noncoding RNAs. *Bioinformatics*, **31**(13), 2199–2201.

TABLE II. Comparison of different transcriptome annotation tools

Tool	Installation steps	User-managed dependencies	Availability	Latest release (year)	Publication
**TransAnnot**	Build[a], Database download, *de novo* assembly (optional)	Plass	GitHub (`https://github.com/soedinglab/transannot.git`), Conda	2024	This publication
**EnTAP**	Dependency installation, Build, .ini file configuration, Databases download	DIAMOND, InterProScan, RSEM, TransDecoder	GitHub (`https://github.com/harta55/EnTAP`)	2023	Hart *et al.* [5]
**Trinotate**	Dependency installation, Build, Database configuration and initialization	BLAST+/Diamond, SQLite, TransDecoder, Trinity[b], HMMER3 (optional), Infernal (optional), signalP (optional), tmhmm2 (optional)	GitHub (`https://github.com/Trinotate/Trinotate`) Docker (`https://hub.docker.com/r/trinityrnaseq/trinotate`), Singularity (`https://data.broadinstitute.org/Trinity/TRINOTATE_SINGULARITY/`), Conda (`https://anaconda.org/bioconda/trinotate`)	2023	None
**Annocript**	Installation[c], MySQL database configuration, Tool configuration, ± UniProt account creation	BLAST+, MySQL	GitHub (`https://github.com/frankMusacchia/Annocript`	2016	Musacchia *et al.* [6]
**dammit**	Installation, Database download	None	GitHub (`https://github.com/dib-lab/dammit`), conda (`https://anaconda.org/bioconda/dammit`)	2019	None
**eggNOG-mapper**	Installation, Database download	None	GitHub (`https://github.com/eggnogdb/eggnog-mapper`), conda (`https://anaconda.org/bioconda/eggnog-mapper`)	2023	Cantalapiedra *et al.* [3]

[a]- If `easytransannot` module is used. [b]- Dependencies are included in Docker or Singularity. [c]- Automatized installation of the dependencies.
